# Synthesis and Biological Evaluation of Novel Thiazolyl-Coumarin Derivatives as Potent Histone Deacetylase Inhibitors with Antifibrotic Activity

**DOI:** 10.3390/molecules24040739

**Published:** 2019-02-19

**Authors:** Viviana Pardo-Jiménez, Patricio Navarrete-Encina, Guillermo Díaz-Araya

**Affiliations:** 1 Laboratory of Advanced Organic Chemistry, Department of Organic Chemistry and Physical Chemistry, Faculty of Chemical and Pharmaceutical Sciences; University of Chile, Santiago 8380000, Chile; pnavarre@ciq.uchile.cl; 2 Laboratory of Molecular Pharmacology, Department of Pharmacological & Toxicological Chemistry, Faculty of Chemical and Pharmaceutical Sciences; University of Chile, Santiago 8380000, Chile; 3 Advanced Center of Chronic Diseases (ACCDiS), Faculty of Chemical and Pharmaceutical Sciences, University of Chile, Santiago 8380000, Chile

**Keywords:** thiazolyl-coumarin, HDACs inhibitor, cardiac fibroblasts, antifibrotic

## Abstract

New histone deacetylases (HDAC) inhibitors with low toxicity to non-cancerous cells, are a prevalent issue at present because these enzymes are actively involved in fibrotic diseases. We designed and synthesized a novel series of thiazolyl-coumarins, substituted at position 6 (R = H, Br, OCH_3_), linked to classic zinc binding groups, such as hydroxamic and carboxylic acid moieties and alternative zinc binding groups such as disulfide and catechol. Their in vitro inhibitory activities against HDACs were evaluated. Disulfide and hydroxamic acid derivatives were the most potent ones. Assays with neonatal rat cardiac fibroblasts demonstrated low cytotoxic effects for all compounds. Regarding the parameters associated to cardiac fibrosis development, the compounds showed antiproliferative effects, and triggered a strong decrease on the expression levels of both α-SMA and procollagen I. In conclusion, the new thiazolyl-coumarin derivatives inhibit HDAC activity and decrease profibrotic effects on cardiac fibroblasts.

## 1. Introduction

Cardiac fibrosis is characterized by the excessive deposition of collagens and extracellular matrix (ECM) proteins that lead to impaired organ function, and is the major factor in the progression of myocardial infarction and heart failure [1]. Cardiac fibroblasts (CFs) play a key role in the homeostatic maintenance of ECM and healing after injury [2]. By the use of transforming growth factor beta1 (TGF–β1) they are differentiated to cardiac myofibroblasts (CMFs), which are cells characterized by increased collagen synthesis, expression and assembly in stress fiber of the α–smooth muscle actin (α–SMA), a contractile protein and marker of CMFs [2]. Recent investigations have shown that the expression and activity of distinct histone deacetylases (HDACs) are strongly correlated to the cardiac fibrosis development. The pattern expression analysis of HDAC1 and HDAC2 in hearts showed that HDAC1 is expressed mainly by fibroblasts/interstitial cells, whereas HDAC2 is more prominent in cardiomyocytes with low levels of expression in fibroblasts/interstitial cells. Moreover, in hearts with heart failure, both HDAC1 and HDAC2 showed strong expression in CFs, although HDAC2 maintained its expression in cardiomyocytes. Together, these data suggest that HDAC1 and HDAC2 are mainly associated with the regulation of the biology of CFs in the heart [3,4,5].

HDACs are enzymes that remove acetyl groups from an ε–N–acetyllysine amino acid on a histone and restore the positive charge to lysine residues [6]. Additionally, non-histone proteins such as transcription factors (e.g., p53, FOXP3), Hsp90, tubulin and other cytoplasmic proteins that play a role in regulatory processes can also be deacetylated by HDACs [7,8,9]. HDACs are a family of 18 enzymes categorized into four classes: class I (HDAC1, 2, 3 and 8); class II (HDAC4, 5, 6, 7, 9 and 10); class III (SIRT1–7), also known as sirtuins; and class IV (HDAC11). Classes I, II and IV are zinc dependent enzymes, and class III are NAD^+^ dependent [10].

HDAC inhibitors (iHDACs) block the access to the active site of HDACs and thus produce hyperacetylation of histones and non-histone proteins. Their effects vary according to cell type and stimulus [11,12]. Hydroxamic acid-based HDAC pan-inhibitors have long been recognized for their antifibrotic action on the heart and, more recently, selective inhibitors of the HDAC class I isoform have been shown to block cardiac fibrosis [13,14,15,16]. In the heart, iHDACs have been shown to reduce interstitial cardiac fibrosis by multiple mechanisms, including inhibition of proliferation and/or migration of CFs, induction of genes that suppress ECM protein synthesis of fibroblast, deletion of proinflammatory signals for fibrosis, and blockade of the endothelial-mesenchymal transition [13,14,15,16].

**Figure 1:**
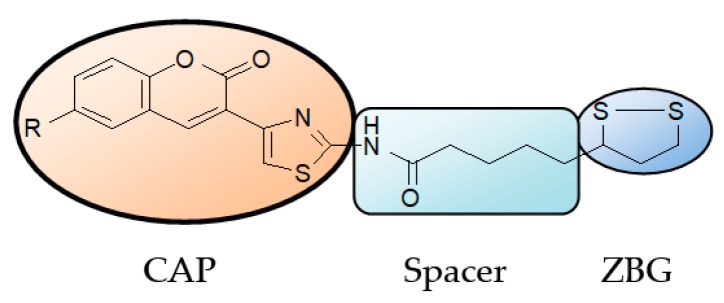
General schematic assembly of the derivatives showing the CAP, spacer and a disulfide ZBG.

iHDACs are compounds either of natural origin or obtained by synthesis and they constitute a very diverse group in terms of structure, biological activity and specificity [17,18]. iHDACs known at present are classified according to their chemical structure in the following groups: hydroxamic acid derivatives, aliphatic acids, benzamides, epoxides, cyclic peptides and sulfonamides containing peptide structures. There are three common characteristics present in these inhibitors: a zinc binding group (ZBG) to coordinate the catalytic metal ion, a hydrophobic spacer that normally has a chain of 5 to 6 methylenes and a surface recognition group (CAP, see Figure 1), which interacts with the outer surface of the enzyme [18,19]. The searching for novel iHDAC compounds with high affinity for these enzymes, but of less structural complexity, low toxicity and easier syntheses remains a challenge for researchers.

Coumarins and their analogues are an important class of compounds of natural and synthetic origin [20]. These compounds belong to the lactone family and are composed of benzene rings condensed onto an α–pyrone ring. In recent years the use of the coumarin ring as a scaffold molecule has occupied an important place in the investigation of new drugs possessing different pharmacological activities such as antibacterial, antiviral, anticancer, anticoagulant, anti-inflammatory, antioxidant and analgesic effects [21,22]. Moreover, coumarin–based benzamides and osthole have been used successfully as CAP in new iHDACs synthesis [23,24]. Coumarin derivatives having various substituted thiazole rings at C-3 exhibit promising biological activities [25]. Recently, syntheses of some new thiazolyl–coumarin derivatives with good anticonvulsivante [26], antimicrobial [27], analgesic and anti-inflammatory activities [28] were also reported. Some thiazolyl–coumarin analogues are found to bepotential anticancer and antimicrobial agents [29], with selective cytotoxicity towards tumor cell lines but not on normal cells [30,31], making them interesting for their evaluation as iHDACs in non-cancer cells.

Another important aspect to consider is that compounds with antifibrotic activity are scarce and nowadays the use of iHDACs has attracted the interest of medicinal chemists due to their participation on avoiding fibrotic disorders [32,33]. This study reports the synthesis, characterization, in vitro inhibitory activity of HDACs and antifibrotic activities of new 3-(2-amino-thiazol-4-yl)-coumarin (CTz) derivatives.

## 2. Results and Discussion

### 2.1. Chemistry

To mimic the structural features of the natural compound trichostatin A (TSA) and the three characteristic sites of the enzyme, i.e., the edge, the tubular pocket and the active site that contains the Zn^2+^ [18,19], the final synthesized compounds are made up of four components: a3-(4-thiazolyl)coumaringroup (CAP), hydrophobic spacer and a ZBG (Figure 1).

#### 2.1.1. Scaffold CAP Synthesis

**Figure 2:**
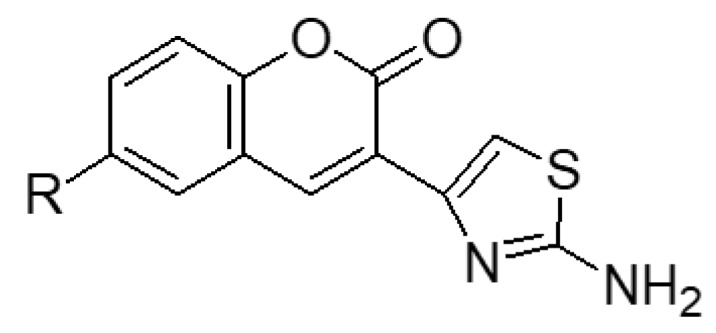
Structure of the CTz derivative scaffold CAP (**4a**, R= H; **4b**,R= Br; **4c**, R= OCH_3_).

The CTz ensemble establishes a scaffold that allows us to obtain derivatives easily by substitutions in the coumarin benzene ring and also allow us to form amide bonds with the amino group at position 2 of the thiazole to subsequently incorporate the spacer. The general structure of the scaffold CAP is shown in Figure 2.

**Scheme 1:**
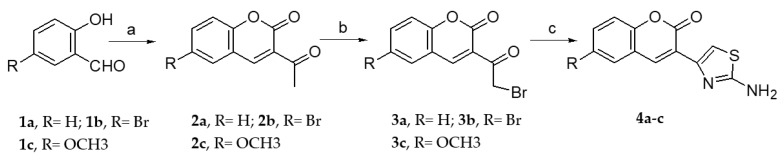
Reagents and conditions: (a) ethyl acetoacetate, piperidine (drops), 50 °C, 0.5–2 h (depending on R) (**2a**, yield= 85%;**2b**,yield= 84%;**2c**, yield= 88%)(b) Br_2_, CHCl_3_/CH_3_COOH, 40–60 °C, 3 h; (**3a**, yield= 80%; **3b**, yield= 86%; **3c**, yield= 66%) (c) (c.1)SC(NH_2_)_2_, EtOH, 78 °C (reflux), 15 min,(c.2) CH_3_COONa, H_2_O, 50 °C (**4a**, yield= 91%; **4b**, yield= 73%;**4c**, yield= 82%).

Starting from 5-substituted-salicylaldehydes **1a**–**c**, compounds **4a**–**c** were synthesized in three steps (Scheme 1).

The first step corresponding to the synthesis of the substituted acetylcoumarin, was carried out by means of a Knoevenagel-type condensation between the corresponding salicylaldehyde (R = H, OCH_3_ and Br) and ethyl acetoacetate in the presence of piperidine as a catalyst. After bromination of the coumarin acetyl group, the last stage of the CAP synthesis was accomplished by using the Hantzsch thiazole synthesis [34]. Fast and easy condensation of bromoacetylcoumarins with thiourea led to the synthesis of derivatives **4a**–**c**. 

#### 2.1.2. Adding the Spacer and the ZBG

**Scheme 2:**
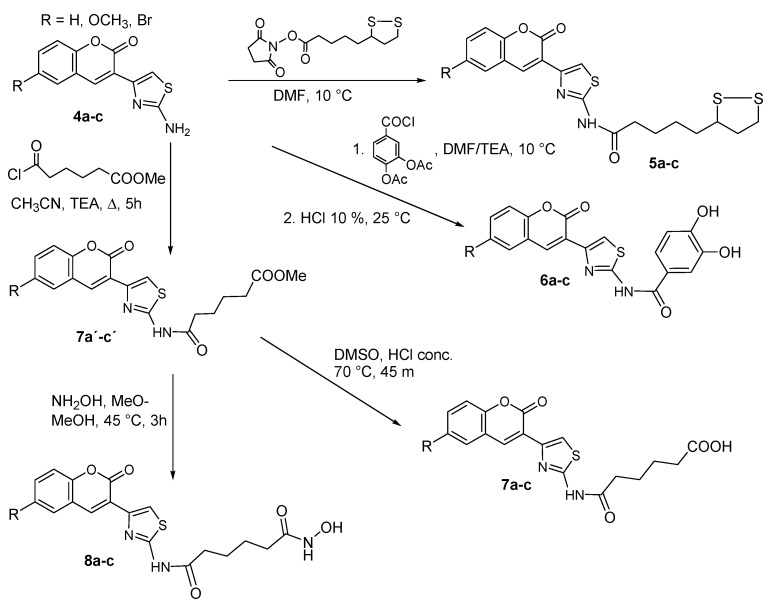
General scheme of the reactions leading to the final derivatives.

In this manuscript we study the use of CTz derivatives linked to a spacer which in turn has a ZBG group chosen for its recognized zinc chelating ability. Four different ZBG groups were tested: disulfide, carboxylic acid, catechol and hydroxamic acid. Synthesis of the final derivatives obtained are described in Scheme 2.

α–Lipoic acid (ALA, 5-(1,2-dithiolan-3-yl) pentanoic acid), has a fivecarbon chain and a disulfide ZBG group. Studies in vitro and in vivo have shown that ALA is able to chelate Zn^2+^, Cu^2+^ and Fe^3+^ ions [35,36,37,38]. The biological effects of ALA are primarily associated with their antioxidant properties [35,36] although it also exhibits antimutagenic, anticarcinogenic activities [37] and produces an increase in normal cell survival [39,40]. 

3,4–Dihydroxybenzoic acid, is a polyphenol broadly distributed in Nature, forming part of the secondary metabolites of a wide variety of plants and fruits [41]. These phenols are strong transition metal chelating agents for Fe^3+^ and Cu^2+^, also forming complexes with Zn^2+^ through their hydroxyl groups [42,43]. Polyphenols are also recognized for their antioxidant activity in addition to exhibiting antimicrobial and anticancer properties [41,44]. 

Valproic and butyric acids are known iHDACs. The inhibitory potency of these compounds is in the low mM range, compared with compounds such as suberoylanilide hydroxamic acid (SAHA) and trapoxin, among others. In most HDAC inhibitory activity studies, short alkyl chain acid derivatives are used [45] and the experimental facts highlight the importance of a voluminous substituent group which must interact with the external surface of the enzyme [18,45]. Therefore, the presence of the CTz as group head could increase the inhibitory potency of the proposed compounds. 

The compounds that contain the hydroxamic acid group as a ligand for Zn^2+^, are those that show the highest inhibitory potency of HDACs [15,45]. This functional group has been widely used in the synthetic strategy of compound inhibitors of matrix metalloproteinases, exhibiting inhibitory concentrations (in vitro) in the nM range [35] but hydroxamic acid derivatives have poor oral bioavailability and are rapidly metabolized in vivo to the corresponding carboxylic acid group [44,46]. In Table 1 we show a summary of the final synthesized and tested compounds.

### 2.2. HDACs Enzyme Inhibition 

The assays of the HDAC inhibitory activity of each of the CTz derivatives were carried out using HeLa cell nuclear extract, HDAC1 and HDAC6 human recombinant as HDAC source. We evaluated the ability of the CTz derivatives, each one in a broad concentration range (10–5000 nM), to inhibit HDACs activity using TSA as positive control. Results were expressed as IC_50_ values and tabulated in Table 2. All compounds showed inhibitory activities against HDACs. Among them, the derivatives **5a**–**c** and **8a**–**c** were the most active inhibitory compounds of HDACs nuclear HeLa cells, HDAC1 and HDAC6 isoforms. Among the **5a**–**c** derivatives, compound **5c** exhibited the highest inhibitory activity with IC_50_ values of 50.0 nM for HeLa cells nuclear HDACs and 39.7 nM for HDAC1, while for HDAC6 IC_50_ value increases to 84.1 nM. In general, compounds belonging to series 1 displayed modest selectivity towards HDAC1 with a 2.3–fold preference for HDAC1 versus HDAC6. It should be noted that the HeLa cells nuclear extract as well as the isoforms HDACs 1 and 6 used in the assay do not present enzymes that can reduce the disulfide to the corresponding thiol; therefore, we suppose that the disulfide acts per se as ZBG. This behavior has been also observed in diallyl disulfide derivatives which have HDAC inhibitory activity without being previously reduced to thiol [47].

Among the derivatives **8a**–**c**, compound **8c** exhibited the highest inhibitory activity with IC_50_ values of 41.5 nM for HeLa cells nuclear HDACs and 46.5 nM for HDAC1, while for HDAC6 the IC_50_ value increases to 62.6 nM. Same tendency was observed for compounds **8a** and **8b**. In comparison with the derivatives of series **5** (**5a**–**c**), those of series **8** (**8a**–**c**) showed less selectivity towards HDAC class I. This result agrees with the literature, since the selectivity of hydroxamic acid group is often questioned in the drug design, because of its high binding affinity to the zinc and other ions [48].

Compounds **6a**–**c** possessing a catechol as ZBG, have lower potency in HDACs inhibition than the compounds of series **5** and **8**, probably because they do not have the spacer length corresponding to 5 to 6 methylenes as described in the literature for optimal interaction with the enzyme [18,19]. It is also observed that the compounds of this series are not selective in the inhibition of class I and class II HADCs. With respect to the compounds **7a**–**c** having a carboxylic acid as ZBG, a weak HDACs inhibitory activity was observed. 

These results demonstrated that CTz group is an effective surface recognition CAP for HDACs inhibition. Since the IC_50_ values of the acid derivatives (4.7–6.1 μM) are higher than the values of short chain fatty acid, such as valproic acid (140 μM) and phenylbutyric acid (620 μM) [49], is indicative that the inclusion of a 6–carbon spacer and CTz is responsible of the improvement in the inhibitory activity.The same is observed with the catecholic derivatives (IC_50_= 477–574 nM) which are more potent inhibitors than catecholic derivatives such as chlorogenic acid (IC_50_= 375 μM) and caffeic acid (2.54 mM) [50]. However, the CTz capping group did not have a significant impact on the selectivity of the HDAC inhibitors for class I and class II.

### 2.3. Cellular Assays

#### 2.3.1. Cytotoxicity Assay

CFs were treated for 48 h with all the new CTz derivatives in a broad concentration range (100 μM^−1^ mM), and their cytotoxicity was then evaluated. In Table 3 cytotoxic concentration 50 (CC_50_) values for each of the synthesized compounds are shown. The data show that all the derivatives have a cytotoxic effect of the same order of magnitude. In each series, the Br derivatives were the ones that caused an increasein cell death, being **8b** the most cytotoxic compound (CC_50_= 72.4 μM). The non-substituted derivatives caused less cell death, being compound **5a** the less cytotoxic one (CC_50_= 200.9 μM). When comparing the different ZBG the series 5 is the least cytotoxic for CFs, while series 8 was the most cytotoxic.

It has been described that HDACs activity has a key role on viability on cancer cells [18,19]. In this study we found no correlation between the HDACs inhibitory activity and cytotoxicity for each series of compounds. These results have also been seen in other studies where iHDACs have been synthesized with alternative ZBG to the hydroxamate, such as mercaptoacetamide and ortho-aminoanilides with higher selectivity towards class I HDACs and less cytotoxic [51].

#### 2.3.2. Cell Proliferation 

CFs were treated for 48 h with 10% fetal bovine serum (FBS), to induce CFs proliferation in presence/absence of each new CTz derivatives in a concentration range of 1–10 μM. In Table 4, the inhibition percentage of cell proliferation for each concentration of the synthesized compounds are shown. All derivatives inhibited CFs proliferation induce by serum. 

The derivatives **5a**–**c** and **8a**–**c** already has a proliferation inhibition percentage of more than 50% at a concentration of 1 μM, the most powerful in inhibition being compounds **5b** and **8b**. While derivatives **6a**–**c** and **7a**–**c** shows a slight inhibition of proliferation at concentrations 1 and 5 μM. These results agree with the results obtained from HDACs inhibitory activity. The derivatives **5a**–**c** and **8a**–**c** shows the highest HDAC inhibitory activity, maintaining the trend of greater activity for these two series in inhibiting the proliferation of CFs. The results also show that at concentrations where serum-induced proliferation is inhibited by more than 50%, these compounds are not cytotoxic.

It is important to highlight that CF proliferation playsa key role in cardiac fibrosis development [48]. Therefore, inhibition of CF proliferation by novel CTz derivatives could be important to prevent cardiac fibrosis. Our results showed that at lower concentration, derivatives **5a**–**c** and **8a**–**c** prevent FBS-induced CFs proliferation. Other iHDACs have been shown to inhibit the proliferation of CFs at similar concentrations found for our derivatives, mocetinostat (1 μM), SAHA (10 μM) and apicidin (3 μM) [3,15]. From these results we can conclude that CTz derivatives (**5a**–**c** and **8a**–**c**) at lower concentration could have important effect on cardiac fibrosis by inhibiting CFs proliferation, a key step in active fibrotic disorders.

#### 2.3.3. Cardiac Fibroblast HDACs Inhibition 

**Figure 3:**
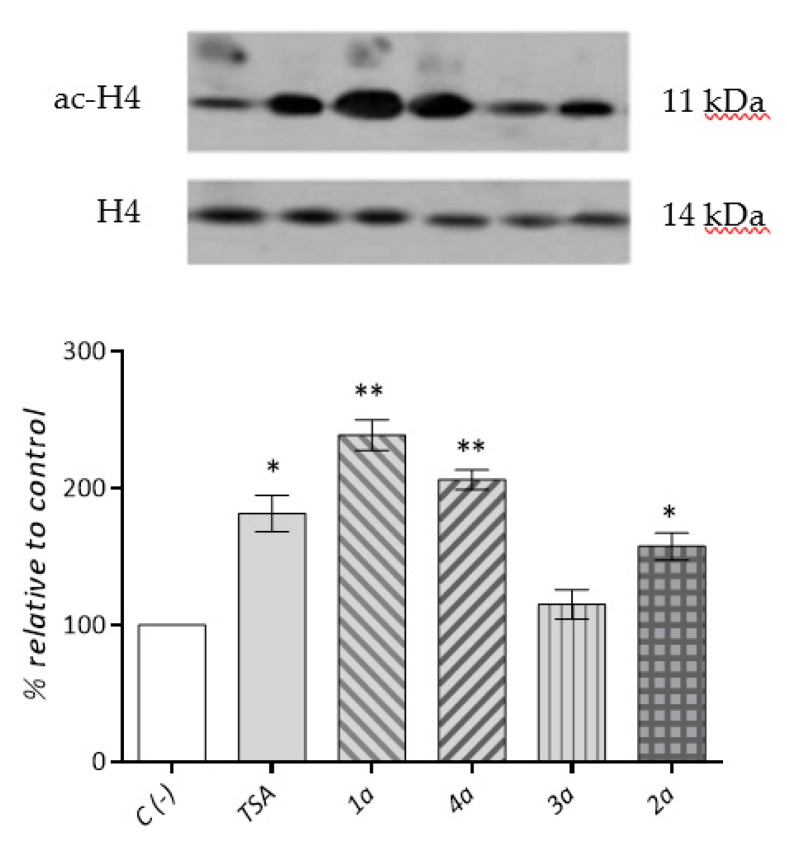
CTz non–substituted derivatives inhibit histone H4 acetylation. CFs were exposed to **5a**, **7a, 6a** and **8a** at 5 μM for 8 h. TSA at 0.1μM was used as positive control. Nuclear extracts were isolated and histone H4 acetylation expression levels were measured by western blot. Histone H4 was used as control load. The results are showed as Mean +/− SD for three independent experiments. **p < 0.01 and *p < 0.05 vs. control.

Our next step was to demonstrate the inhibitory effect on histone deacetylase activity in CFs. For this purpose, CFs of neonatal rats were incubated in the presence/absence of the **5a**, **6a**, **7a** and **8a** derivatives, and the expression of histone H4–acetylated was measured using the western blot technique. Inhibition of HDACs was studied at non-cytotoxic concentrations and TSA was used as a control and also for comparative purposes. In the upper panel of Figure 3, a representative image of the expression of histone H4–acetylated is exhibited, whereas in the lower panel we show the corresponding graphical analysis. The results demonstrate that at anuclear level, CFs expresses detectable levels of H4–acetylated protein. Also, we observed that the **5a**, **6a** and **8a** derivatives increase in a statistically significant manner the histone H4–acetylated expression, while the derivative **7a** had no effect.

Derivatives **5a** and **8a** showed higher histone H4-acetylated expression levels. These results were complementary and coincident within vitro HDAC inhibition and show that the derivatives increased histone H4–acetylated expression levels, indicative of HDACs inhibition in CFs. These results are quite significant, since no cytotoxic effects were observed at working concentration. 

#### 2.3.4. Cardiac Fibroblast α-SMA Expression Levels 

It has been indicated that HDACs are important in CFs–to–CMFs differentiation, an important features of cardiac fibrosis development. Thus our objective was to demonstrate that in the CFs the CTz derivatives reduce α–SMA expression levels and prevent those induced by TGF–β1, therefore inhibiting the differentiation process.

**Figure 4:**
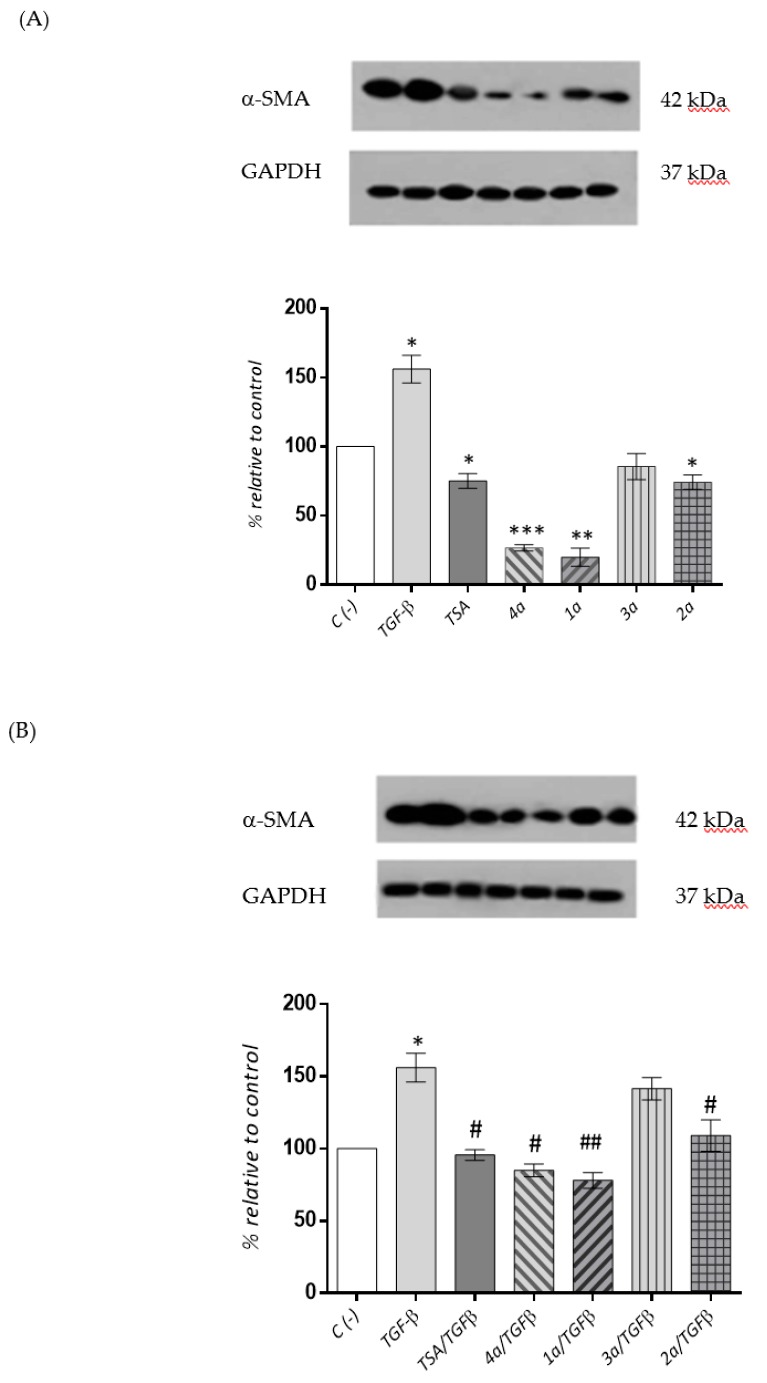
CTz non–substituted inhibit α-SMA expression in cardiac fibroblasts. (**A**) CFs were exposed to **5a**, **6a**, **7a** and **8a** at 5 μM for 48 h. TSA (0.1 μM) and TGF–β1 (5 μg/mL) were used as positive control. (**B**) CFs were exposed to **5a**, **6a**, **7a** and **8a** at 5 μM for 48 h in presence of TGF–β1 (5 μg/mL). α-SMA expression levels were measured by western blot. GAPDH was used as control load. The results are showed as Mean +/− SD for three independent experiments. *p < 0.05, **p < 0.01 and ***p < 0.001 vs. control, #*p* < 0.05 and ##*p* < 0.01 vs. TGF-β1.

For this purpose, a fixed concentration of **5a**, **6a**, **7a** and **8a** derivatives in the presence/absence of TGF–β1 (a strong inducer of CFs–to–CMFs differentiation), was studied, and α–SMA expression levels were measured by using the western blot technique. TSA was used as a control and also for comparative purposes. In the upper panels of Figure 4 (A and B), representative photographs of α-SMA expression level and glyceraldehyde 3-phosphate dehydrogenase(GAPDH) (used as charge control) are exhibited, while in the lower panel the graphic analyses are shown. In Figure 4A, the results show significant α–SMA expression levels in CFs, and TGF–β1 significantly increased α-SMA expression levels with respect to control levels. In absence of TGF–β1, **5a**, **6a** and **8a** derivatives decreased in a statistically significant manner α-SMA expression levels being **5a** and **8a** derivatives the compounds that strongly decreased α-SMA expression levels, while compound **7a** had no effect. In Figure 4B it can be seen that TGF-β1 significantly increased the expression levels of α-SMA with respect to control levels. Pretreatment of CFs with **5a**, **6a**, **8a** and TSA produce a decrease close to control levels on α-SMA expression levels induced by TGF–β1.

#### 2.3.5. Cardiac Fibroblast Procollagen Type I Expression Levels

**Figure 5:**
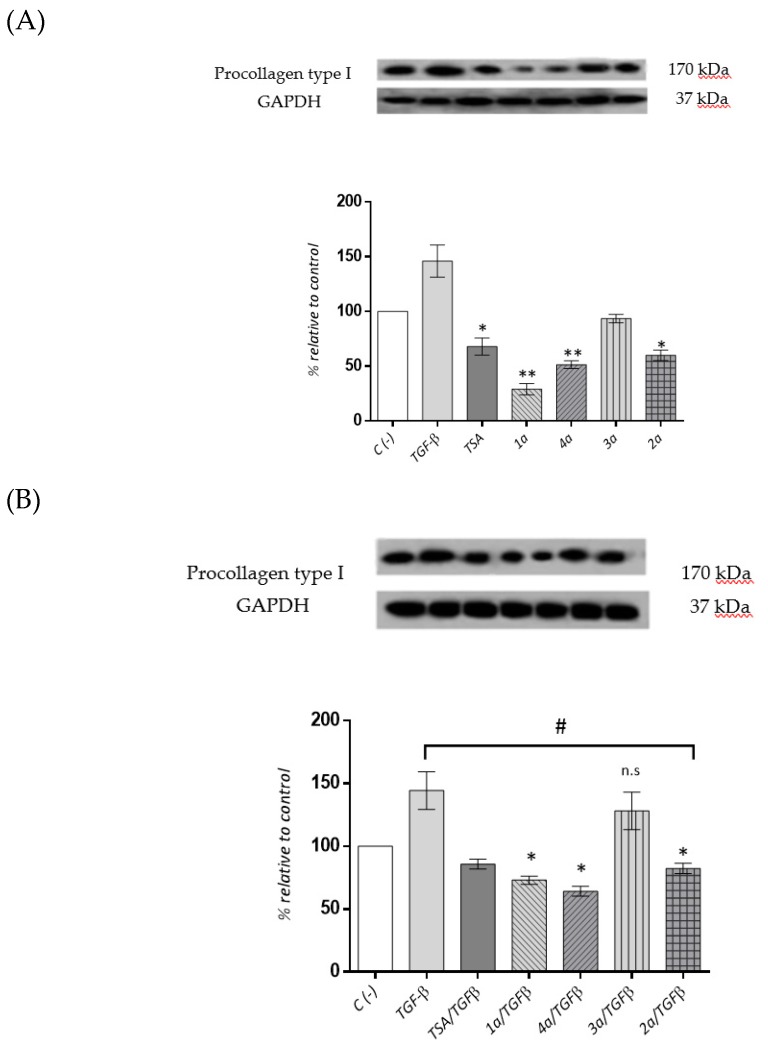
CTz non–substituted inhibit procollagen type I expression levels in cardiac fibroblasts. (**A**) CFs were exposed to **5a**, **6a**, **7a** and **8a** at 5 μM for 48 h.TSA (0.1μM) and TGF–β1 (5 μg/mL) were used as positive control. (**B**) CFs were exposed to **5a**, **6a**, **7a** and **8a** at 5 μM for 48 h in presence of TGF–β1. Procollagen type I expression levels were measured by western blot. GAPDH was used as control load. The results are showed as mean +/− SD for three independent experiments. *p < 0.05 and **p < 0.01 vs. control, #*p* < 0.05 vs. TGF–β1.

In CFs collagen type I secretion is a hallmark of cardiac fibrosis development, which is regulated by HDACs. Thus our objective was to demonstrate that in CFs the CTz derivatives reduce procollagen type I expression levels and prevent those induced by TGF–β1. For this purpose, a fixed concentration of **5a**, **6a**, **7a** and **8a** derivatives in presence/absence of TGF-β1 were studied, and procollagen type I expression levels were measured by using the western blot technique. TSA was used as control and also for comparative purposes. In Figure 5 (A and B), in the upper panel representative photographs of procollagen type I expression level and GAPDH (used as charge control) are shown, whereas in the lower panel the graphic analyses are observed. In Figure 5A, it can be seen that there is significant procollagen type I expression levels in CFs, and TGF–β1 significantly increased procollagen type I expression levels with respect to control. In absence of TGF–β1, **5a**, **6a**, and **8a** derivatives decreased in a statistically significant manner procollagen type I expression levels with respect to control, being **5a** derivative the most potent compound, while compound **7a** had no effect. In Figure 5B, the results show that TGF–β1 significantly increased procollagen type I expression levels with respect to control levels. Pretreatment of CFs with **5a**, **6a**, and **8a** derivatives and TSA decreased, close to control levels, procollagen type I expression levels induced by TGF–β1.

α–SMA is a protein marker of CFs–to–CMF differentiation, a key process in cardiac fibrosis development, whereas collagen type I is the main extracellular matrix protein secreted by CFs, and it is the main ECM protein involved in cardiac fibrosis development. TGF–β1 is the main growth factor responsible for this differentiation process, and also for collagen synthesis and secretion. Our results showed that **5a**, **6a** and **8a** derivatives decreased both α–SMA and procollagen type I expression levels, either under basal conditions (not stimulated) or under TGF–β1 stimulation, being **5a** and **8a** the most potent inhibitors. These results are consistent with our previous results in which we showed that **5a** and **8a** derivatives were the best HDAC activity inhibitors and those that mostly increased the histone H4-acetylated expression levels. In general terms, our results are consistent with those reported in cardiac and lung fibroblasts where TSA and SAHA inhibit the expression of contractile protein α–SMA [52] as well as, those found in cultured rat CFs, in which TSA blocks TGF–β1–induced collagen synthesis [52,53]. However, we cannot rule out the possibility that our CTz compounds as iHDACs prevent α–SMA and procollagen expression levels through additional mechanisms [54].

Collectively, our results highlight the importance of the disulfide–derivatives and hydroxamic acid ones as the most potent iHDACs. The disadvantages of hydroxamic acids are the biological limitations associated with poor HDACs selectivity and cytotoxicity. 

### 2.4. ADMET Analysis

We cannot ignore the influence of the physicochemical characteristics of each synthesized compounds on the results obtained in the assays carried out in CFs, for this reason a theoretical analysis of the absorption, distribution, metabolism, excretion and toxicity (ADMET) parameters of each CTz was carried out.

The ADMET parameters of CTz derivatives were measured using QikProp software (Table 5). Compounds of the series **5**,**6** and **8** have partition coefficient (QplogPo/w) values in the permeable range 1.8–5.0. The Caco-2 cell permeability (QPPCaco) was in the permissible range of 29–1177. The percentage human oral absorption for the series 1 compounds is 100%, while for the others compounds ranged from 58 to 79%. Compounds belonging to series 7 are out of range of acceptable partition coefficient values. In consequence this may explain the poor effects obtained on CFs assays. 

Parameters of the compounds belonging to series **5,6** and **8** were all within the acceptable range defined for human use and therefore these compounds may exhibit significant pharmacokinetic and drug likeness properties.

## 3. Experimental Section

### 3.1. Chemistry

#### 3.1.1. General Information

Nuclear magnetic spectra (^1^H-NMR, 300 MHz and ^13^C-NMR, 75.47MHz) were recorded on an Avance DRX 300 spectrometer (Bruker, Billerica, MA, USA) at ambient temperature. Fourier-transform infrared spectroscopy (FTIR) spectra were recorded with a Bruker IFS 55 spectrophotometer in the 600–4000 cm^−1^ range. Matrix-assisted laser desorption/ionization mass spectrometry (MALDI-MS) mass spectra were recorded on a MALDI-time of flight (MALDI-TOF) Microflexsystem (Bruker Daltonics GmbH, Leipzig, Germany) in positive ion mode. Electrospray ionization mass spectra (ESI-MS) were obtained on an electrospray-ion trap type ESI-IT Esquire 4000 mass spectrometer (Bruker Daltonik GmbH, Leipzig, Germany). Melting points were measured on a Electrothermal IA9000 Series Digital Melting Point Apparatus (Rochford, UK) and are uncorrected. Silica gel 60 (0.040–0.063 mm) for column chromatography (Merck, Darmstadt, Germany) was employed. All solvents were purified by routine techniques prior to use.

2-Hydroxybenzaldehyde p.a., ethyl acetoacetate (AcOEt) p.a., acetone p.a., glacial acetic acid p.a., 98% sulfuric acid, sodium acetate p.a., acetic anhydride p.a., chloroform p.a., dimethylsulfoxide (DMSO) p.a., ethanol p.a., sodium hydroxide p.a., sodium sulfate p.a., toluene p.a., were purchased from Merck. 5-methoxy-salicylaldehyde p.a., 5-bromo-salicylaldehyde p.a., 99.9% acetone d6, 3,4-dihydroxybenzoic acid., methyl adipoyl chloride p.a., 99.9% chloroform d6, 99.9% dimethylsulfoxide d6, were from Sigma-Aldrich (St Louis, MO, USA). Methanol p.a., *N,N*-dimethylformamide (DMF), p.a., 37% hydrochloric acid p.a., were from J.T.Baker (Phillipsburg, NJ, USA). Nitrogen 99.995% extra pure was purchased from Linde Gas (Santiago, R.M. Chile).

#### 3.1.2. General Synthesis of 6-substituted-3-acetylcoumarins **2a**–**c**

These derivatives were synthesized in accordance with the Knoevenagel procedure [55]. In a 100 mL Erlenmeyer flask, 82 mmol of the corresponding salicylaldehyde **1a**–**c**, 82 mmol of ethyl acetoacetate and three drops of piperidine as catalyst were added. The solution is stirred at 50 °C. After 15 min the appearance of a yellow precipitate was observed and heating was continued for another 30 min. The reaction conditions varied depending on the substituent, with the 5-methoxy-salicylaldehyde the reaction was carried out at room temperature for 1 h, while using the 5-bromo-salicylaldehyde the reaction was refluxed with ethanol for 2 h. The products of the reactions were monitored by thin layer chromatography (silica gel 60 F254), using EtOAc/hexane 1:2 mixtures as the mobile phase. All pure coumarins were obtained by recrystallization from ethanol. The compounds were characterized by their melting points, ^1^H-NMR, ^13^C-NMR and FTIR.

#### 3.1.3. General Synthesis of 3-bromoacetylcoumarins **3a**–**c**

6-Substituted-3-acetylcoumarins **2a**–**c** (15.9 mmol) were dissolved in chloroform (40 mL) and glacial acetic acid (10 mL), then Br_2_ (15.9 mmol) was carefully added dropwise while stirring the solution at room temperature. Once the addition of Br_2_ was finished, the mixture was heated between 40–60 °C thus facilitating the emission of hydrobromic acid fumes. After 3 h of reaction, precipitation of 3-bromoacetylcoumarin was observed. The solution was allowed to cool to room temperature, filtered and the precipitate washed with three 10 mL portions of cold ethanol. All brominated derivatives were recrystallized from an 80:20 ethanol/chloroform mixture and characterized by their melting points, ^1^H-NMR, ^13^C-NMR, and FTIR [56,57].

#### 3.1.4. General Synthesis of 6-substituted-3-(2-amino-thiazol-4-yl)-coumarins **4a**–**c**

To an ethanol solution (150 mL) of 6-substituted-3-bromoacetilcoumarin **3a**–**c** (6.3 mmol) at 50 °C an equimolar amount of thiourea was added with vigorous stirring and maintained at 70 °C. Ten min after the addition of thiourea, the mixture turned to an intense orange-yellow color and further precipitation of a yellow solid was observed. After evaporation of the solvent to 50% of the initial volume, the mixture was cooled, the solid filtered out and washed with cold ethanol. The yellow solid was dissolved in 200 mL of a hot solution of 10% ammonium acetate in water, stirred for 10 min and then allowed to cool to room temperature. The solids corresponding to **4a**–**c** were filtered. All compounds were recrystallized from ethanol and characterized by their melting points, ^1^H-NMR, ^13^C-NMR and FTIR.

#### 3.1.5. Synthesis of *N*-hydroxysuccinimide (NHS)-lipoate (**9**)

**Figure 6:**
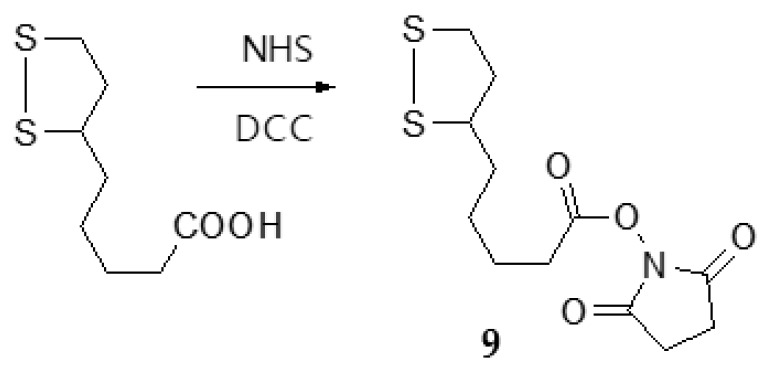
Synthesis of *N*-hydroxysuccinimide (NHS)-lipoate.

Lipoic acid (1.0 g, 5 mmol) was dissolved in dry DMF (10 mL) in a 50 mL Erlenmayer flask, the solution was cooled to 10 °C in an ice bath and NHS (0.6 g, 5 mmol) was added. The mixture was stirred for 15 min and dicyclohexylcarbodiimide (DCC, 1.0 g, 5 mmol) was added. The solution was stirred for 3 h in an ice bath and further maintained for 9 h at room temperature. The white solid dicyclohexylurea (DCU) was filtered off. The solution was cooled to 8 °C for 5 h and the remaining DCU precipitate was filtered again. The procedure was repeated until no DCU crystals were observed. The solution in DMF containing the activated lipoic (Figure 6) acid was used directly in the formation of compounds **5a**–**c.**

#### 3.1.6. General Synthesis of 6-substituted-5-[1,2]-dithiolan-3-yl-pentanoic acid [4-(2-oxo-2*H*-chromen-3-yl)-thiazol-2-yl]-amides **5a**–**c**

**Figure 7:**
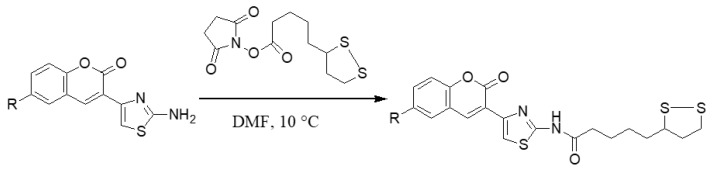
General synthesis of 6-substituted-5-[1,2]-dithiolan-3-yl-pentanoic acid [4-(2-oxo-2*H*-chromen-3-yl)-thiazol-2-yl]-amides.

Compounds **4a**–**c** (4.80 mmol) were added to compound **9** (4.35 mmol) dissolved in dry DMF (25 mL) at 10 °C. The mixture was stirred for 12 h at room temperature (Figure 7). The DMF solution was slowly poured into 100 mL H_2_O/ice. A pale yellow solid precipitated. The solid was filtered off and washed with H_2_O and then added to a cold concentrated HCl (37%) solution to dissolve the unreacted **4a**–**c**. The remaining solid was filtered and neutralized with a 10% aqueous sodium bicarbonate solution. The obtained precipitate was purified by using a Merck 60 silica gel column (70–230mesh) and EtOAc/chloroform 1:1 as mobile phase. The yellow color solids were characterized by their melting point, ^1^H-NMR, ^13^C-NMR, FTIR and mass spectrometry.

#### 3.1.7. General Synthesis of 6-substituted-*N*-[4-(2-oxo-2*H*-chromen-3-yl)-thiazol-2-yl]-3,4-dihydroxy-acetylbenzamide Coumarins **6a′**–**c′**

**Figure 8:**
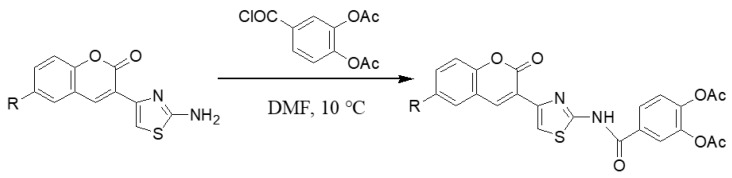
General synthesis of 6-substituted *N*-[4-(2-oxo-2*H*-chromen-3-yl)-thiazol-2-yl]-3,4-dihydroxy-acetylbenzamide coumarins.

3,4-Diacetylbenzoic acid chloride (1.2 mmol) was dissolved in dry DMF (10 mL), cooled to 10 °C and **1a**–**c** (R = H, OCH_3_ and Br, 1.7 mmol) dissolved in dry DMF (6 mL) was added dropwise. After 20 min of stirring, triethylamine (Et_3_N, 1 mL) was added, and the mixture stirred for 16 h from 10 °C to room temperature (Figure 8). The formed salt was filtered off and the DMF solution was slowly poured into 50 mL H_2_O/ice forming a yellow colored precipitate. The solid was filtered and washed with H_2_O, then it was added to aqueous cold HCl (37%) to dissolve the compounds **4a**–**c** that did not react, the remaining solid was filtered and neutralized with a solution of sodium bicarbonate. The precipitate obtained was purified by using a Merck 60 silica gel column (70–230 mesh), using the mobile phase EtOAc/dichloromethane 2:1 obtaining a yellow-brown solid which was characterized by their melting point, ^1^H-NMR, ^13^C-NMR, FTIR and mass spectrometry. 

#### 3.1.8. General Synthesis of 6-substituted *N*-[4-(2-oxo-2*H*-chromen-3-yl)-thiazol-2-yl]-3,4-dihydroxy-benzamide Coumarins **6a**–**c**

Each derivative **6a′**–**c′** (1.2 mmol) was dissolved in DMF (10 mL) and added to 20% HCl (10 mL). The mixture was stirred at room temperature for 30 min. The formed precipitate was washed with 10% sodium bicarbonate solution and purified using a Merck 60 silica gel column (70–230mesh), using as mobile phase EtOAc/dichloromethane 2:1. Yellow-brown solids corresponding to compounds **6a**–**c**, were obtained, recrystallized in ethanol and characterized by their melting points, ^1^H-NMR, ^13^C-NMR and FTIR.

#### 3.1.9. General Synthesis of 6-substituted methyl 5-[4-(2-oxo-2*H*-chromen-3-yl)-thiazol-2-yl-carbamoyl]-pentanoate Coumarins **7a′–c′**

**Figure 9:**
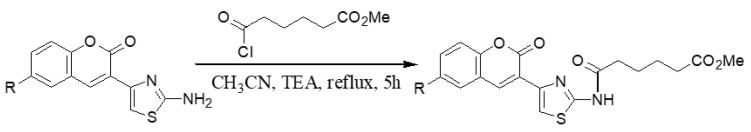
General synthesis of 6-substituted methyl 5-[4-(2-oxo-2*H*-chromen-3-yl)-thiazol-2-yl-carbamoyl]-pentanoate coumarins.

In a 100 mL round bottom flask each compound **4a**–**c** (6 mmol) was dissolved in dry acetonitrile (30 mL), adipoyl acid monomethyl ester chloride (6 mmol) and Et_3_N (1 mL) were added and the mixture was stirred at reflux for 5 h (Figure 9). A pale yellow solid precipitated, was filtered off and washed with cold acetonitrile and further purified by recrystallization from glacial acetic acid and column chromatography using silica gel Merck 60 (70–230 mesh) and EtOAc/dichloromethane 1:1 as mobile phase. The solid obtained was characterized by their melting point, ^1^H-NMR, ^13^C-NMR, FTIR and mass spectrometry. Mild hydrolysis of the methyl esters yields the corresponding pentanoic acid derivatives **7a**–**c**. 

#### 3.1.10. General synthesis of 6-substituted5-[4-(2-oxo-2*H*-chromen-3-yl)-thiazol-2-yl-carbamoyl]-pentanoicacidcoumarins **7a**–**c**

Each compound **7a′**–**c′** (2.6 mmol) was dissolved in DMSO (10 mL) and slowly added to a solution of 37% HCl (20 mL). The mixture was heated to 70 °C with stirring for 45 min. Subsequently the solution was cooled to form a precipitate, which was filtered off and washed with cold water. The products were purified by using Merck 60 silica gel column (70–230mesh), using the mobile phase EtOAc/dichloromethane 1:3. The whitish yellow solids were characterized by their melting point, FTIR and ^1^H-NMR. Due to its insolubility it was not possible to characterize these acid derivatives by ^13^C-NMR or MS.

#### 3.1.11. General synthesis of 6-substituted-5-[4-(2-oxo-2*H*-chromen-3-yl)-thiazol-2-yl-carbamoyl] –pentanoate hydroxamic acid derivatives **8a**–**c**

**Figure 10:**
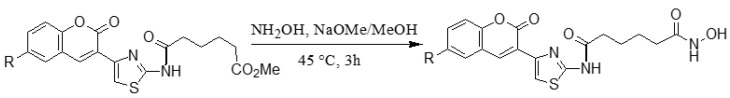
General synthesis of 6-substituted-5-[4-(2-oxo-2*H*-chromen-3-yl)-thiazol-2-yl-carbamoyl]–pentanoate hydroxamic acid derivatives.

Each compound **7a′**–**c′** (3.9 mmol) was added to a solution with hydroxylamine (3.9 mmol) in 10% sodium methoxide in methanol (100 mL). The mixture was heated to 45 °C for 3 h (Figure 10). Methanol was removed using a rotavapor at room temperature. The residue was neutralized with a cold solution of 10% HCl in methanol. The NaCl formed was filtered off and the methanol was removed again with a rotary evaporator, forming a waxy yellow precipitate. The products were purified by using Merck 60 silica gel column (70–230 mesh), using the mobile phase methanol/dichloromethane 1:3. The yellow solids obtained were characterized by their melting point, ^1^H-NMR, ^13^C-NMR, FTIR and mass spectrometry.

#### 3.1.12. Physical Characterization of the Main Precursors and Final Synthesized Compounds

*3-(2-Aminothiazol-4-yl)-coumarin* (**4a**). Yellow crystals, m.p.: 228–230 °C. Yield: 91%. FTIR (cm^−1^): 3311 (-NH_2_); 3148 (aromatic); 1700 (O-C=O, lactone); 1643 (-C=N-); 1602 (-C=C-). ^1^H-NMR (δ, DMSO-d_6_): 5.00 (s, 2H, -NH-); 7.20–7.70 (m, 4H, -ArH); 7.85 (s, 1H, -CH=, thiazole); 8.72 (s, 1H, -CH =, coumarin). ^13^C-NMR (δ, DMSO-d_6_):112.0; 115.8; 117.5; 124.6; 125.0; 127.1; 133.2; 137.4; 145.3; 154.4; 157.3; 170.2. 

*3-(2-Aminothiazol-4-yl)-6-bromocoumarin* (**4b**). Yellow-pale crystals, m.p.: 224 °C. Yield: 73%. FTIR (cm^−1^): 3341 (-NH_2_); 3157 (aromatic); 1710 (O-C=O, lactone). ^1^H-NMR (δ, DMSO-d_6_): 5.10 (s, 1 H, -NH-); 7.20 (d, 1H, *J* = 8.4 Hz, -ArH); 7.80 (d, 1H, *J* = 8.47 Hz, -ArH); 7.95 (s, 1H, -ArH); 7.85 (s, 1H, -CH=, thiazole); 8.92 (s, 1H, -CH=, coumarin). ^13^C-NMR (δ, DMSO-d_6_): 112.5; 115.9; 118.0; 121.2; 125.3; 128.4; 135.3; 138.6; 146.1; 155.8; 158.7; 172.2.

*3-(2-Aminothiazol-4-yl)-6-methoxycoumarin* (**4c**). Yellow-orange crystals, m.p.: 201–203 °C. Yield: 82%. FTIR (cm^−1^): 3300 (-NH_2_); 3133 (aromatic); 1705 (O-C=O, lactone). ^1^H-NMR (δ, DMSO-d_6_): 3.74 (s, 3H, -OCH_3_); 5.00 (s, 1H, -NH-); 7.08 (s, 1H, -ArH_5_); 7.4 (d, 1H, *J* = 8.5 Hz, -ArH); 7.7 (d, 1H, *J* = 8.5Hz, -ArH); 7.80 (s, 1H, -CH=, thiazole); 8.7 (s, 1H, -CH=, coumarin). ^13^C-NMR (δ, DMSO-d_6_): 56.3; 108.5; 113. 6; 115.0; 115.8; 117.9; 128.5; 133.2; 135.3; 143.2; 152.0; 154.8; 168.9. 

*5-[1,2]-Dithiolan-3-yl-N-[4-(2-oxo-2H-chromen-3-yl)-thiazol-2-yl]-pentanamide* (**5a**). Pale yellow solid, m.p.: 91–92 °C. Yield: 80.6%. FTIR (cm^−1^): 3155 (aromatic); 2931 (-CH-); 1726 (O-C = O, lactone); 1648 (-C= N-); 1540 (-C=C-). ^1^H-NMR (δ, DMSO-d_6_): 1.60 (m, 4H, -CH_2_-); 1.94 (m, 2H, -CH_2_-); 2.42 (m, 4H, -CH_2_-); 2.67 (m, 2H, -CH_2_-); 3.65 (m, 1H, -CH=); 7.3–7.8 (m, 4H, -ArH); 7.5 (s, 1H, -CH=, thiazole); 8.55 (s, 1H, -CH=, coumarin). ^13^C-NMR (δ, DMSO-d_6_): 25.5; 27.0; 33.2; 37.6; 39.3; 40.2; 55.1; 107.0; 117.3; 118.1; 122.4; 124.2; 127.5; 133.7; 139.3; 148.2; 154.5; 157.6; 160.4; 175.1. MALDI-MS theoretical *m*/*z*: 433.0714; *m*/*z* experimental: 433.0694; error (ppm): −4.6. ESI-MS: *m*/*z*: 433.0 [M + H]^+^; 431.0 [M − H]^−^. 306 [M-H_2_O + C_3_H_6_S_2_ + H]^+^; 245 [M-CO-C_4_H_7_-C_3_H_5_S_2_ + H]^+^; 225 [M-H_2_O + CO + C_4_H_9_-C_3_H_5_S_2_ + H]^+^; 207 n.d. 397 [M-H_2_S − H]^−^. Molecular formula: C_20_H_20_O_3_N_2_S_3_. Molecular weight (monoisotopic): 432.0636 g/mol

*5-[1,2]-Dithiolan-3-yl-N-[4-(6-bromo-2-oxo-2H-chromen-3-yl)-thiazol-2-yl]-pentanamide* (**5b**). Pale yellow solid, m.p.: 150–153 °C. Yield: 70.5%. FTIR (cm^−1^): 3197 (aromatic); 2945 (-CH-); 1697 (O-C = O, lactone); 1625 (-C = N-); 1538 (-C = C-). ^1^H-NMR (δ, DMSO-d_6_):1.56 (m, 4 H, -CH_2_ -); 1.88 (m, 2H, -CH_2_-); 2.42 (m, 4H, -CH_2_-); 2.67 (m, 2H, -CH_2_-); 3.62 (m, 1H, -CH=); 7.15 (s, 1H, -NH-); 7.20–7.62 (m, 3H, -ArH); 7.53 (s, 1H, -CH=, thiazole); 8.49 (s, 1H, -CH=, coumarin). ^13^C-NMR(δ,DMSO-d_6_): 25.7; 27.3; 33.0; 36.9; 38.7; 40.8; 56.1; 107.8; 117.6; 119.0; 122.6; 124.7; 128.5; 134.2; 140.2; 149.5; 155.5; 158.4; 160.9; 176.3. MALDI-MS: theoretical *m/z*: 510.9819; *m/z*experimental: 510.9796; error (ppm): -4.5. ESI-MS: *m/z*: 513.0 [M + H]^+^; 511.0 [M + H]^+^; 511 [M − H]^−^. 397 [M-HBr + H2S + H]^+^; 325 [M-CO-C_4_H_7_-C_3_H_5_S_2_ + H]^+^; 306 [M-HBr + H_2_O + C_3_H_6_S_2_ + H]^+^; 189 [M-Br-C_9_H_4_O_2_-C_3_HNS-NH_2_ + H]^+^. 498 n.d; 396 [M-HBr + H_2_S + H]^+^; 323 [M-CO-C_4_H_7_-C_3_H_5_S_2_ + H]^+^; 189 [M-Br-C_9_H_4_O_2_-C_3_HNS-NH_2_+H]^+^. 477 [M-H_2_S − H]^−^. Molecular Formula: C_20_H_19_O_3_N_2_S_3_Br. Molecular weight (monoisotopic): 509.9741 g/mol.

*5-[1,2]-Dithiolan-3-yl-N-[4-(6-methoxy-2-oxo-2H-chromen-3-yl)-thiazol-2-yl]-pentanamide* (**5c**). Yellow solid, m.p.: 83–84 °C. Yield: 70.5%. FTIR (cm^−1^): 3083 (aromatic); 2922 (-CH-); 1702 (O-C = O, lactone); 1640 (-C = N-); 1556 (-C=C-). ^1^H-NMR (δ, DMSO-d_6_): 1.55 (m, 4H, -CH_2_-); 1.88 (m, 2H, -CH_2_-); 2.41 (m, 4H, -CH_2_-); 2.68 (m, 2H, -CH_2_-); 3.62 (m, 1H, -CH=); 7.15 (s, 1H, -NH-); 7.4 (d, 1H, *J* = 8.5 Hz, -ArH) 7.55 (s, 1H, -CH=, thiazole); 7.7 (d, 1H, *J* = 8.5 Hz, -ArH); 8.1 (s, 1H, -ArH); 8.45 (s, 1H, -CH=, coumarin). ^13^C-NMR (δ, DMSO-d_6_): 25.3; 27.15; 31.7; 36.3; 39.5; 40.9; 54.3 55.1; 108.2; 110.5; 117.1; 117.4; 117.7; 119.1; 139.7; 146.3; 146.4; 153.3; 154.5; 158.2; 177.1. MALDI-MS theoretical *m*/*z*: 463.0820; *m*/*z*experimental: 463.0797; error (ppm): −5.0. ESI-MS: *m*/*z*: 463.0 [M + H]^+^; n.d [M − H]^−^. 398 [M-C_3_OH + H_2_S_2_ + H]^+^; 275 [M-CO-C_4_H_7_-C_3_H_5_S_2_ + H]^+^. Molecular formula: C_21_H_22_O_4_N_2_S_3_. Molecular weight (monoisotopic): 462.0742 g/mol.

*N-[4-(2-Oxo-2 H-chromen-3-yl) -thiazol-2-yl]-3,4-dihydroxyacetyl-benzamide* (**6a′**). Pale yellow solid, m.p.: 175–177 °C. Yield: 75%. FTIR (cm^−1^): 3064 (aromatic); 2926 (-CH-); 1707 (O-C=O, lactone); 1609 (-C=N-); 1485 (-C=C-). ^1^H-NMR (δ, DMSO-d_6_):3.00 (s, 3H, -CH_3_); 3.17 (s, 3H, -CH_3_); 7.18 (s, 1H, -NH); 7.30–7.98 (m, 7H, -ArH); 7.85 (s, 1H, -CH=, thiazole); 8.73 (s, 1H, -CH=, coumarin). ^13^C-NMR (δ, DMSO-d_6_): 25.3 (d); 109.2; 113.9; 116.3; 119.7 (d); 121.0; 125.2; 129.2; 131.9; 132.0; 138.5; 139.3; 137.4; 143.7; 144.5; 152.6; 152.8; 157.3; 159.4 (d); 167.9; 173.6. MALDI-MS theoretical *m*/*z*: 465.0756; *m*/*z* experimental: 465.0737; error (ppm): −4.0. ESI-MS: *m*/*z*: 465.0 [M + H]^+^; n.d [M − H]^−^. 424 [M-CH_3_CHO +H]^+^; 387 [M-H_2_O + HO-COCH_3_ + H]^+^; 258 n.d; 136 [M-H-C_9_H_4_O_2_-C_3_H-NS-NH-CO + CO-CH_2_O + H]^+^. Molecular Formula: C_23_H_16_O_7_N_2_S. Molecular weight (monoisotopic): 464.0678 g/mol.

*N-[4-(6-Bromo-2-oxo-2H-chromen-3-yl)-thiazol-2-yl]-3,4-dihydroxyacetyl-benzamide* (**6b′**). Pale yellow solid, m.p.: 214–217 °C. Yield: 70%. FTIR (cm^−1^): 3068 (aromatic); 2931 (-CH-); 1727 (O-C=O, lactone); 1631 (-C=N-); 1504 (-C=C-). ^1^H-NMR (δ, DMSO-_d6_):3.05 (s, 3H, -CH_3_); 3.19 (s, 3H, -CH_3_); 7.18 (s, 1H, -NH); 7.35-8.00 (m, 6H, -ArH); 7.92 (s, 1H, -CH=, thiazole); 8.91 (s, 1H, -CH=, coumarin). ^13^C-NMR (δ, DMSO-d_6_):26.4 (d); 109.5; 114.0; 116.5; 119.8; 119.4121.0; 126.6; 130.0; 133.2; 133.4; 139.0; 140.3; 144.1; 144.8; 153.4; 154.1; 157.9; 162.4 (d); 169.8; 177.3. MALDI-MS theoretical *m/z*: 542.9862; experimental *m*/*z*: 542.9839; error (ppm): −4.2. ESI-MS: *m*/*z*: n.d. [M + H]^+^; n.d. [M − H]. Due to the low intensity of the signals, fragmentation characterization at low resolution could not be performed. Molecular Formula: C_23_H_15_O_7_N_2_SBr. Molecular weight (monoisotopic): 541.9783 g/mol.

*N-[4-(6-Methoxy-2-oxo-2H-chromen-3-yl)-thiazol-2-yl]-3,4-dihydroxyacetyl-benzamide* (**6c′**). Yellow solid, m.p.: 118–120 °C. Yield: 68.2%.FTIR (cm^-1^): 3116 (aromatic); 2927 (-CH-); 1714 (O-C=O, lactone); 1611 (-C=N-); 1572 (-C=C-).^1^H-NMR (δ, DMSO-d_6_):3.00 (s, 3H, -CH_3_); 3.16 (s, 3H, -CH_3_); 3.83 (s, 3H, -OCH_3_); 7.00–8.10 (m, 6H, -ArH); 7.80 (s, 1H, -CH =, thiazole); 8.41 (s, 1H, -NH); 8.72 (s, 1H, -CH=, coumarin). ^13^C-NMR (δ, DMSO-d_6_):25.3 (d); 58.8; 108.1; 112.7; 115.3; 118.6; 120.9; 124.5; 128.7; 130.8; 131.7; 137.5; 138.3; 142.5; 144.3; 151.3; 152.0; 156.1; 158.5 (d); 166.6; 172.8. MALDI-MS theoretical *m/z*: 495.0862; experimental *m/z*: 495.0840; error (ppm): −4.4. ESI-MS: *m*/*z*: 495.0 [M + H]^+^; 493.0 [M − H]^−^. 450 [M-CH_3_ CHO + H]^+^; 401 [M-H_2_O + CO_2_ + CH_3_OH + H]^+^; 372 [M-H_2_O + HO-COCH_3_ + CH_3_CHO + H]^+^; 270 [M-CH_2_O + C_6_H_4_-(O-CO-CH_3_)_2_]^+^; 452 [M-CH_2_CO− H]^−^; 479 [M-CH_3_− H]^−^. Molecular formula: C_24_H_18_O_8_N_2_S. Molecular weight (monoisotopic): 494.0784 g/mol.

*5-[4-(2-Oxo-2H-chromen-3-yl)-thiazol-2-yl-carbamoyl]-pentanoic acid methyl ester* (**7a′**). Pale yellow solid, m.p.: 165–166 °C. Yield: 75%. FTIR (cm^−1^): 3185 (aromatic); 2981 (-CH-); 1687 (O-C = O, lactone); 1643 (-C= N-); 1539 (-C=C-). ^1^H-NMR (δ, DMSO-d_6_):1.60 (m, 4H, -CH_2_-); 2.35 (t, 2H, -CH_2_-); 2.49 (t, 2H, -CH_2_-) 3.59 (s, 3H, -OCH_3_); 7.05–7.47 (m, 4H, Ar-H); 7.51 (s, 1H, -CH=, thiazole); 8.2 (s, 1H, -NH); 8.48 (s, 1H, -CH=, coumarin). ^13^C-NMR (δ, DMSO-d_6_):24.7; 25.7; 32.2; 33.7; 49.9; 109.1; 116.3; 119.5; 120.4; 125.0; 129.1; 132.2; 138.7; 152.7; 154.1; 159.2; 168.2; 169.8; 173.0. MALDI-MS theoretical *m*/*z:* 387.1015; experimental *m/z*: 387.0996; error (ppm): −4.9. ESI-MS: *m*/*z*: 387.0 [M + H]^+^; 385.0 [M − H]^−^. 355 [M-CH_3_OH + H]^+^; 311 [M-H_2_O + COOCH_2_ + H]^+^; 271 [M-C_4_H_9_-COOCH_3_ + H]^+^; 245 [M-CO-C_4_H_7_-COOCH_3_ + H]^+^. 353 [M-CH_3_OH − H]^−^; 310 [M-H_2_O + COOCH_2_− H]^−^; 243 [M-CO-C_4_H_7_-COOCH_3_− H]^−^. Molecular formula: C_19_H_18_O_5_N_2_S. Molecular weight (monoisotopic): 386.0936 g/mol.

*5-[4-(6-Bromo-2-oxo-2H-chromen-3-yl)-thiazol-2-yl-carbamoyl]-pentanoic acid methyl ester* (**7b′**). Yellow solid, m.p.: 182–185 °C. Yield: 61.4%. FTIR (cm^−1^): 3067 (aromatic); 2943 (-CH-); 1704 (O-C=O, lactone); 1639 (-C= N-); 1536 (-C= C-). ^1^H-NMR (δ, DMSO-d_6_):1.70 (m, 4H, -CH_2_-); 2.38 (t, 2H, -CH_2_-); 2.56 (t, 2H, -CH_2_-); 3.61 (s, 3H, -OCH_3_); 7.30 (d, 1H, *J* = 8.4 Hz, Ar-H); 7.80 (d, 1H, *J* = 8.4 Hz, Ar-H); 7.83 (s, 1H, Ar-H); 8.00 (s, 1H-CH=, thiazole); 8.92 (s, 1H, -CH =, coumarin). ^13^C-NMR (δ, DMSO-d_6_):24.9; 25.8; 32.9; 34.5; 51.8; 110.0; 119.7; 120.3; 121.4; 125.5; 129.8; 135.2; 140.6; 153.7; 155.6; 160.0; 170.3; 173.1; 173.9. MALDI-MS theoretical *m/z*: 465.0120; experimental *m/z*: 465.0103; error (ppm): −3.7. ESI-MS: *m/z*: 467.0 [M + H]^+^; 465.0 [M − H]^+^; 463.0 [M − H]^−^. 435 [M-CH_3_OH + H]^+^; 325 [M-CO-C_4_H_7_-COOCH_3_ + H]^+^; 433 [M-CH_3_OH + H]^+^; 409 n.d; 323 [M-CO-C_4_H_7_-COOCH_3_ + H]^+^; 431 [M-CH_3_OH-H]^−^; 321 [M-CO-C_4_H_7_-COOCH_3_− H]^−^. Molecular formula: C_19_H_17_O_5_N_2_SBr. Molecular weight (monoisotopic): 464. 0042 g/mol.

*5-[4-(6-Methoxy-2-oxo-2H-chromen-3-yl)-thiazol-2-yl-carbamoyl]-pentanoic acid methyl ester* (**7c′**). Yellow solid, m.p.: 163–164 °C. Yield: 64%. FTIR (cm^−1^): 3188 (aromatic); 2935 (-CH-); 1705 (O-C=O, lactone); 1614 (-C = N-); 1540 (-C=C-). ^1^H-NMR (δ, DMSO-d_6_):1.75 (m, 4H, -CH_2_-); 2.36 (m, 2H, -CH_2_-); 2.50 (m, 2H, -CH_2_-); 3.68 (s, 3H, -OCH_3_); 6.96 (s, 1H, -NH); 7.06 (d, 1H, *J* = 8.5 Hz -ArH); 7.30 (d, 1H, *J* = 8.5 Hz -ArH); 7.75 (s, 1H, -CH =, thiazole); 8.23 (s, 1H, -ArH); 8.44 (s, 1H, -CH =, coumarin). ^13^C-NMR (δ, DMSO-_d6_):24.5; 25.3; 33.1; 35.0; 49.3; 55.1; 109.0; 116.0; 116.4; 117.0; 119.5; 124.0; 138.5; 151.1; 152.5; 158.6; 166.3; 169.8; 173.0. MALDI-MS theoretical *m/z*: 417.1120; experimental *m/z*: 417.1102; error (ppm): −4.3. ESI-MS: *m*/*z*: 417.0 [M + H]^+^; 415.0 [M − H]^−^. 385 [M-CH_3_OH + H]^+^; 341 [M-H_2_O + COOCH_2_ + H]^+^; 301 [M-C_4_H_9_-COOCH_3_ + H]^+^; 275 [M-CO-C_4_H_7_-COOCH_3_ + H]^+^. 383 [M-CH_3_OH − H]^−^; 273 [M-CO-C_4_H_7_-COOCH_3_− H]^−^. Molecular formula: C_20_H_20_O_6_N_2_S. Molecular weight (monoisotopic): 416.1042 g/mol.

*5-[4-(2-Oxo-2H-chromen-3-yl)-thiazol-2-yl-carbamoyl]-N-hydroxypentanamide* (**8a**). Pale yellow solid, m.p.: 150–152 °C (d). Yield: 64%. FTIR (cm^−1^): 3057 (aromatic); 2925 (-CH-); 1699 (O-C=O, lactone); 1640 (-C=N-); 1539 (-C=C-).^1^H-NMR (δ, DMSO-d_6_): 1.50 (m, 4 H, -CH_2_-); 2.02 (m, 4H, -CH_2_-); 5.50 (s, 1H, -CONH-); 6.50-7.30 (m, 4H, Ar-H); 7.97 (s, 1H, -CH =, thiazole); 8.50 (s, 1H, -CONHOH); 8.60 (s, 1H, -CH=, coumarin). ^13^C-NMR (δ, DMSO-d_6_):24.7; 25.7; 32.2; 33.7; 49.9; 109.1; 116.3; 119.5; 120.4; 125.0; 129.1; 132.2; 138.7; 152.7; 154.1; 159.2; 168.2; 169.8; 173.0. MALDI-MS theoretical m/z: 388.0967; experimental m/z: 388.0953; error (ppm): −3.6. ESI-MS: *m*/*z:* 388.0 [M + H]^+^; 386.0 [M − H]^−^. 370 [M-H_2_O + H]^+^; 355 [M-NH_2_OH + H]^+^; 271 [M-C_4_H_9_-CONHOH + H]^+^; 245 [M-CO-C_4_H_7_-CONHOH + H]^+^. 353 [M-NH_2_OH − H]^−^; 259 n.d.; 243 [M- CO-C_4_H_7_-CONHOH − H]^−^; 141 [M-H-C_9_H_4_O_2_-C_3_HNSNH_2_− H]^−^. Molecular Formula: C_18_H_17_O_5_N_3_S. Molecular weight (monoisotopic): 387.0889 g/mol.

*5-[4-(6-Bromo-2-oxo-2H-chromen-3-yl)-thiazol-2-yl-carbamoyl]-N-hydroxypentanamide* (**8b**). Pale yellow solid, m.p.: 106–110 °C (d). Yield: 57%. FTIR (cm^−1^): 3032 (aromatic); 2951 (-CH-); 1698 (O-C=O, lactone); 1648 (-C=N-); 1537 (-C=C-). ^1^H-NMR (δ, DMSO-_d6_):1.50 (m, 4H, -CH2-); 2.50 (m, 4H, -CH_2_-); 7.24 (s, 1H, -CONH-); 7.52 (s, 1H, -CH=, thiazole); 7.39 (d, 1H, *J* = 9.0 Hz, ArH); 7.40 (s, 1H, ArH); 7.73 (d, 1H, *J* = 9.0 Hz, ArH); 8.10 (s, 1H, -CONHOH); 8.50 (s, 1H, -CH= coumarin). ^13^C-NMR (δ, DMSO-d_6_): 31.5; 32.4; 34.4 (d); 110.1; 116.8; 118.5; 121.7; 121.8; 131.3; 134.1; 131.0; 134.1; 137.1; 143.5; 151.5; 158.7; 168.0. Due to the insolubility of the compound it was not possible to perform mass spectrometry analysis. Molecular Formula: C_18_H_16_O_7_N_2_SBr. 

*5-[4-(6-Methoxy-2-oxo-2H-chromen-3-yl)-thiazol-2-yl-carbamoyl]-N-hydroxypentanamide* (**8c**). Pale yellow solid, m.p.: 125–127 °C (d). Yield: 68%.FTIR (cm^-1^): 3136 (aromatic); 2916 (-CH-); 1721 (O-C=O, lactone); 1631 (-C=N-); 1555 (-C=C-).1H-NMR (δ, DMSO-d_6_):1.56 (m, 4H, -CH_2_-); 1.97 (m, 4H, -CH_2_-); 3.82 (s, 3H, -OCH_3_); 7.20 (d, 1H, *J* = 9.0 Hz, ArH); 7.53 (s, 1H, ArH); 7.34 (s, 1H, -CONH-); 7.38 (d, 1H, *J* = 9.0 Hz, ArH); 7.98 (s, 1H, -CH =, thiazole); 8.50 (s, 1H, -CONHOH); 8.53 (s, 1H, -CH= coumarin). ^13^C-NMR (δ, DMSO-d_6_): 24.8; 25.2; 32.6; 35.2; 56.3; 109.4; 111.1; 111.5; 117.4; 119.7; 120.1; 121.0; 139.0; 147.0; 155.3; 159.2; 168.3; 169.3; 172.1. MALDI-MS theoretical m/z: 418.1073; m/z experimental: 418.1053; error (ppm): −4.8. ESI-MS: *m*/*z*: 418.0 [M + H]^+^; 416.0 [M − H]^−^. 400 [M-H_2_O + H]^+^; 385 [M-CH_3_OH + H]^+^; 301 [M-C_4_H_9_-CONHOH + H]^+^; 275 [M-CO-C_4_H_7_-CONHOH + H]^+^. 383 [M-CH_3_OH − H]^−^; 288 n.d.; 273 [M-CO-C_4_H_7_-CONHOH − H]^−^; 141 [M-H-CH_3_O-C_9_H_4_O_2_-C_3_HNS-NH_2_− H]^−^. Molecular Formula: C_19_H_19_O_6_N_3_S. Molecular weight (monoisotopic): 417.0995 g/mol.

### 3.2. Biology 

#### 3.2.1. Reagents

Fetal bovine serum (FBS), trypsin/ethylenediaminetetraacetic acid (EDTA), molecular weight standard and organic and inorganic compounds were purchased from Merck (Darmstadt, Germany). Reagents for enhanced chemiluminescence (ECL) were purchased from PerkinElmer Life Sciences (Boston, MA, USA). Sterile plastic material was obtained from Corning Inc. (Corning, NY, USA). Primary anti-histone H4–acetylated antibody and total histone H4 were purchased from R & D Systems (Minneapolis, MN, USA). Primary anti–collagen type I and anti–α–SMA were purchased from Sigma-Aldrich (St. Louis, MO, USA). Primary anti–GAPDH antibody and secondary antibodies conjugated to horseradish peroxidase (HRP) were obtained at Santa Cruz Biotechnology (Dallas, TX, USA). Alamar Blue^®^ from Invitrogen was purchased to Thermo Fisher (Waltham, MA, USA). 

#### 3.2.2. Cell Culture.

Sprague Dawley rats were obtained from the Animal Breeding Facility of the School of Chemical and Pharmaceutical Sciences at the University of Chile. All studies followed the Guide for the Care and Use of Laboratory Animals published by the US National Institutes of Health (NIH Publication 8^th^, 2011), and experimental protocols were approved by the University of Chile Institutional Ethics Review Committee. CF were isolated from of 2 or 3–day–old Sprague-Dawley rats and cultured as described previously [58]. Briefly, the neonatal rats were decapitated and their hearts were extracted under aseptic environment. Atria were removed and ventricles were cut into small pieces (~1–2 mm) for posterior colagenase II digestion. The digestion yield was separated by 10 min centrifugation at 89×*g*. The pellet was resuspended in 10 mL of Dulbecco’s Modified Eagle’s Medium/Nutrient F-12 (DMEM-F12) supplemented with 10% FBS and antibiotics (100 μg/mL streptomycin and 100 units/mL penicillin) and cultured in a humid atmosphere of 5% CO_2_ and 95% O_2_ at 37°C until confluence (5 days). The purity of the CFs population was assessed through the expression of several markers. CFs had positive staining against vimentin (Santa Cruz Biotechnology), while being negative against sarcomeric actin and desmin (Sigma-Aldrich). Experiments were performed on cells at passage 1. Cells were cultured in 35-mm well plates and serum starved for 24 h prior to stimulation.

#### 3.2.3. Cytotoxicity and Cell Proliferation Assay

To evaluate cytotoxicity, CFs were seeded in 12 microwell plates at a ratio of 2 × 10^4^ cell/cm^2^, cultured in DMEM–F12 medium + 10% FBS, which was subsequently replaced by DMEM–F12. After 24 h of serum starvation derivatives were applied at proper times and concentrations in accordance to experimental procedures. Cell viability assays were carried out using Alamar Blue^®^, a nontoxic, cell permeable non fluorescent compound that is blue in color. Viable cells reduce resazurin to resorufin, which produces very bright red fluorescence which is used as a quantitative cell viability measure. 10 μL of 0.2 mg/mL Alamar Blue^®^ was added into each well followed by incubation for 24 h. The fluorescence of Alamar Blue^®^ was read using microplate reader at 570 excitation and emission to 595 nm. Cell survival was expressed as percentage over the untreated cells. To evaluate CFs proliferation assay, cells were seeded as mentioned before, and after serum starvation, they were stimulated by 10% fetal bovine serum (FBS), in presence/absence of CTz derivatives for 24 h, at concentrations according to experiment. Cell proliferation was quantified by Alamar Blue^®^, according to protocol mentioned before. Cell proliferation was expressed as percentage over the control FBS treated cells. 

#### 3.2.4. HDACs Inhibitory Activity Assay

The inhibitory activity of the synthesized compounds against HDACs Hela cells nuclear extract, HDAC1 (class I) and HDAC6 (class II) were measured utilizing the Fluorometric Drug Discovery Assay Kit BML-AK500-0001 (HeLa cells nuclear extract), BML-AK511 (HDAC1), and BML-AK516 (HDAC6) (Enzo Life Science, Farmingdale, NY, USA). The HDACs fluorometric substrate and assay buffer were added to HeLa nuclear extracts and also to HDACs 1 and 2 substrates in a 96–wellplate and incubated at 37 °C for 30 min. TSA at 5 nM served as the positive control. The fluorophore produced was detected in a plate spectrofluorimeter, with an excitation of 360 nm and an emission of 460 nm. The assay was performed according to the manufacturer’s instructions, at various concentrations of the synthesized compounds.

#### 3.2.5. Preparation of the Cell Lysate

For the analysis of the protein expression of histone H4–acetylated (ac–H4) and total H4 histone, a number of 1 × 10^6^ of CFs seeded in 60 mm plates was used, which were incubated with the inhibitory compounds at a concentration of 10 μM, in the absence/presence of TGF–β1(5ng/mL) for 4 h. Subsequently, total histones were extracted by the technique of saline precipitation (high salt extraction). Briefly, CFs were scraped from the plates with 1 mL of cold PBS transferring their contents to Eppendorf tubes to be centrifuged for 60 s at 1500 rpm at 4 °C and re–suspended in 400 μL of cold buffer A (10 mM 4-(2-hydroxyethyl)-1-piperazineethanesulfonic acid-KOH (HEPES-KOH) adjusted to pH 7.9, 1.5 mM MgCl_2_, 10 mM KCl, 0.5 mM dithiothreitol and 0.2 mM phenylmethylsulfonyl fluoride (PMSF). The suspension is left on ice for 10 min and then placed in the vortex for 10 s. The samples were centrifuged for 60 s, subsequently discarding the supernatant fraction. The obtained pellet was re-suspended in 60 μL of cold C buffer (20 mM HEPES-KOH adjusted to pH 7.9, 25% glycerol, 420 mM NaCl, 1.5 mM MgCl_2_, 0.2 mM EDTA, dithiothreitol 0.5 mM and 0.2 mM PMSF) and placed on ice for 20 min for saline precipitation. Cell debris is removed by centrifugation for 120 s at 1500 rpm at 4 °C and the supernatant fraction, which contains the DNA binding proteins, is stored at minus 20 °C. For the analysis of α–SMA and procollagen type I expression levels, 3×10^5^ CFs seeded in 35 mm plates were used, which were incubated together with inhibitor compounds at a 5 μM, in the absence/presence of TGF–β1 (5 ng/mL) for 48 h. To obtain the total proteins, the CFs were washed three times with cold PBS, then scraped and lysed with 50 μL of radio inmuno-precipitation assay (RIPA) lysis buffer (10 mM Tris–HCl pH 7.2, 5 mM EDTA, 150 mM NaCl, Triton X–100 1%, sodium dodecyl sulfate (SDS) 0.1%, deoxycholate 1%, leupeptin 2 μg/mL, aprotinin 10mM, PMSF 1mM and Na_3_VO_4_ 100μM). The solution was centrifuged for 15 min at 10,000 rpm and at 4 °C. The supernatant fraction containing the total proteins was stored at minus 20 °C. The concentration of proteins obtained was determined by spectrophotometry at 570 nm, using the Bradford method (Bio-Rad, Hercules, CA, USA).

#### 3.2.6. Western Blot Analysis 

CFs proteins (25 μg), were subjected to 10% sodium dodecyl sulfate-polyacrylamide gel electrophoresis and finally transferred onto a nitrocellulose membrane (Millipore, Billerica, MA, USA). The membrane was washed twice with tris–buffered saline solution containing 0.1% Tween 20 (TBST). After being blocked with TBST containing 5% nonfat milk for 40 min, the membrane was incubated with primary antibodies in TBST/1% nonfat milk at 4 °C overnight. The membrane was washed three times with TBST for a total of 15 min and then incubated with primary antibodies (ac–H4, H4 total, α-SMA, procollagen type I and GAPDH). Then the membrane was incubated with goat anti–rabbit or anti-mouse IgG antibodies conjugated with horseradish (diluted 1:1000) for 1 h at room temperature. Signals were developed with ECL reagents and exposure to photographic film.

#### 3.2.7. Prediction of ADMET Properties

The ADMET properties of the thiazolyl–coumarin derivatives as drug candidate were predicted using the software QikProp (Schrödinger Release2018-4: QikProp, Schrödinger, LLC, New York, NY, USA, 2018).

## 4. Conclusions

Synthesis and evaluation of different ZBGs linked to thiazolyl–coumarin moieties shows that the series **5** and series **8** derivatives are potent HDAC inhibitors. All derivatives inhibit HDAC enzymatic activity (to a greater or lesser degree), suggesting that the thiazolyl–coumarin group is an effective surface recognition CAP. All compounds at lower concentration display diminished cytotoxicity. Regarding the parameters associated to cardiac fibrosis development, compounds **5a** and **8a** show significant inhibition on CFs proliferation at 1 μM and also showed better decrease of procollagen type I and α–SMA expression levels.

These results confirm that series **5** and **8** derivatives could be considered as leads to develop drugs with strong antifibrotic activity and are consistent with published reports where HDAC inhibitors could be useful pharmacological tools to prevent cardiac fibrosis development. Finally, the advantage of thiazolyl–coumarin disulfide over hydroxamic acid derivatives is their low cytotoxicity and better ADMET parameters. 

## Figures and Tables

**Table 1:** Summary of the synthesized and tested compounds (CTz derivatives).

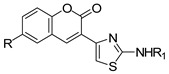			
Compound	R_1_	R	Yield (%)
**5a**	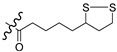	H	80.6
**5b**	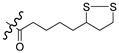	Br	70.5
**5c**	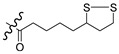	OCH_3_	72.5
**6a**	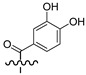	H	71.0
**6b**	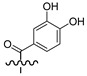	Br	82.0
**6c**	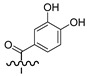	OCH_3_	78.0
**7a**	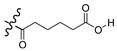	H	81.0
**7b**	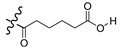	Br	85.0
**7c**	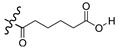	OCH_3_	77.0
**8a**	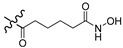	H	64.0
**8b**	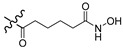	Br	57.0
**8c**	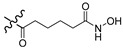	OCH_3_	68.0

**Table 2:** Half maximal inhibitory concentration of HDACs activity.

Compounds	Nuclear HeLa Cells Extract IC_50_ (nM)	Class I	Class II
		HDAC1 IC_50_ (nM)	HDAC6 IC_50_ (nM)
**5a**	66.7 ± 2.9	60.4 ± 1.7	153.4 ± 5.3
**5b**	62.1 ± 2.5	47.6 ± 2.3	122.8 ± 5.1
**5c**	50.0 ± 1.7	39.7 ± 1.3	84.1 ± 2.9
**6a**	573.5 ± 24.4	422.8 ± 30.2	276.1 ± 20.9
**6b**	508.7 ± 23.3	612.7 ± 32.8	449.5 ± 20.7
**6c**	476.7 ± 27.5	504.4 ± 31.4	580.5 ± 27.5
**7a**	6100 ± 390	7689 ± 328	5589 ± 184
**7b**	5400 ± 400	4666 ± 362	6336 ± 466
**7c**	4700 ± 310	3382 ± 302	2427 ± 157
**8a**	66.9 ± 1.8	50.6 ± 2.1	98.3 ± 3.5
**8b**	57.1 ± 4.5	45.4 ± 2.6	80.2 ± 3.7
**8c**	41.5 ± 2.7	46.5 ± 2.2	62.6 ± 2.3
**TSA**	5.0 ± 0.12	2.1 ± 0.06	48.0 ± 4.4

**Table 3:** Half maximal inhibitory concentration cytotoxic.(CC_50_) of new CTz derivatives.

Compound	CC_50_ (μM)
**5a**	200.9 ± 20.3
**5b**	127.2 ± 19.2
**5c**	170.7 ± 6.1
**6a**	140.2 ± 11.7
**6b**	102.2 ± 10.3
**6c**	124.5 ± 12.8
**7a**	111.4 ± 4.4
**7b**	80.0 ± 23.6
**7c**	99.5 ±6.3
**8a**	90.5 ± 5.1
**8b**	72.5 ± 11.6
**8c**	86.5 ± 5.9

**Table 4:** Inhibition percentage of cell proliferation.

Compound	1 μM	5 μM	10 μM
**5a**	57.3 ± 6.7	70.7 ± 7.1	92.8 ± 6.4
**5b**	71.5 ± 3.8	89.1 ± 9.5	96.4 ± 4.7
**5c**	67.0 ± 8.3	83.2 ± 6.8	91.2 ± 3.2
**6a**	3.0 ± 3.9	35.0 ± 1.9	60.6 ± 1.9
**6b**	15.8 ± 3.6	40.0± 1.8	64.1 ± 1.7
**6c**	10.9 ± 3.6	42.2 ± 1.8	64.9 ± 2.3
**7a**	0.5 ± 0.1	26.8 ± 5.9	45.0 ± 5.1
**7b**	2.4 ± 1.1	36.1 ± 5.4	48.0 ± 4.8
**7c**	6.0 ± 0.4	33.0 ± 4.1	48.8 ± 5.2
**8a**	59.4 ± 6.1	79.9 ± 9.7	94.4 ± 3.8
**8b**	72.7 ± 3.9	94.4 ± 7.0	104.0 ± 2.8
**8c**	69.7 ± 9.1	81.8 ± 5.5	97.7 ± 4.7

**Table 5:** ADMET parameters prediction for the thiazolyl–coumarin compounds using QikProp.

Compounds	QlogPo/w ^a^	QPPCaco ^b^	QPlogBB ^c^	Percent Human Oral Absorption ^d^	QPlogKp ^e^	#metab ^f^
**5a**	4.4	1177	−0.5	100	−1.8	2
**5b**	5.0	1173	−0.4	100	−2.0	2
**5c**	4.5	1171	−0.6	100	−1.9	3
**6a**	1.8	134	−1.5	75	−3.4	3
**6b**	2.4	134	−1.4	78	−3.6	3
**6c**	1.9	129	−1.6	76	−3.6	4
**7a**	0.3	38	−2.1	57	−4.1	3
**7b**	0.8	37	−2.0	60	−4.3	3
**7c**	0.4	38	−2.2	58	−4.2	4
**8a**	2.3	29	−1.8	66	−3.8	3
**8b**	2.9	29	−1.7	70	−4.0	3
**8c**	2.4	29	−1.9	67	−3.9	4

^a^ Predicted octanol/water partition coefficient (acceptable range: 2.0–6.5).^b^ Predicted Caco-2 cell permeability in nm/s (acceptable range: <25 is poor, >500 is great).^c^ Predicted brain/blood partition coefficient (Concern value is −3.0 to −1.2).^d^ Predicted human oral absorption on 0–100% scale (acceptable range: <25% is poor, >80% is high).^e^ Predicted skin permeability, log Kp (acceptable range: −8.0 to −1.0).^f^ Number of likely metabolic reactions (acceptable range: 1–8).
